# Tackle injury epidemiology and performance in rugby league – narrative synthesis

**DOI:** 10.17159/2078-516X/2021/v33i1a9313

**Published:** 2021-03-02

**Authors:** N Burger, B Jones, S Hendricks

**Affiliations:** 1 Division of Exercise Science and Sports Medicine, Department of Human Biology, University of Cape Town, Rondebosch, South Africa; 2 Department of Public & Occupational Health and the EMGO Institute for Health and Care Research, VU University Medical Center, Amsterdam, The Netherlands; 3 Carnegie Applied Rugby Research (CARR) Centre, Carnegie School of Sport, Leeds Beckett University, Leeds, UK; 4 Leeds Rhinos Rugby League Club, Leeds, UK; 5 England Performance Unit, The Rugby Football League, Leeds, UK; 6 School of Science and Technology, University of New England, NSW, Australia

**Keywords:** tackler, ball carrier, injury prevention, aetiology, tackling

## Abstract

**Background:**

In rugby league (RL), the ability to repeatedly engage in the tackle, whether as a ball carrier or tackler, is essential for team success and player performance. It is also the leading cause of injury, with over 90% of total injuries occurring during the tackle in professional and amateur cohorts. To effectively reduce the risk of injury and optimise performance, establishing the extent of the ‘problem’, through injury surveillance or descriptive performance studies is required.

**Objective:**

The purpose of this narrative synthesis was to systematically search and synthesise tackle injury epidemiology and tackle performance frequency in RL. To achieve this objective, a systematic review was conducted.

**Methods:**

The search was limited to English-only articles published between January 1995 and October 2018. Based on the search criteria, a total of 53 studies were found: 32 focused on tackle injury epidemiology (nine cases studies) and 21 focused on tackle frequency.

**Results:**

In general, over 600 tackles may occur during an RL match. Tackle injury frequencies (both overall and time-loss injuries) ranged between 47%–94% at the professional level, and between 38%–96% for the lower levels of play. A greater proportion of injuries occurring in professional RL are severe time-loss injuries when compared to lower levels of play. Most time-loss and overall injuries occur to players who are tackled, i.e., ball carriers, across all levels of play.

**Conclusion:**

This narrative synthesis will facilitate tackle injury prevention and performance research in RL, and act as a reference document for coaches and practitioners.

Rugby league (RL) is an intermittent, high-intensity, collision-based team sport.^[1–3]^ During a typical match, played over the course of 60–80 minutes and depending on the level of play, players physically engage each other to compete for ball possession and territory.^[4]^ This dynamic and physical contest is known as the tackle and can occur up to 700 times during a match.^[2]^ As such, the ability to repeatedly engage in the tackle, whether as a ball carrier or tackler, is essential for team success and player performance.^[5]^ It is also the leading cause of injury, with over 90% of total injuries occurring during the tackle in some professional and amateur cohorts.^[6,7]^

To effectively reduce the risk of injury and optimise performance, following a sport injury prevention or sport performance process model such as the Translating Research into Injury Prevention Practice (TRIPP) model^[8]^ (involving six stages) or the Applied Research Model for the Sport Sciences (ARMSS)^[9]^ (involving eight stages), is recommended. These models outline a sequence of stages that need to be completed to ensure the uptake and sustainability of an injury prevention or performance initiative in the ‘real world’.^[8]^ In these models, the first stage proposes establishing the extent of the ‘problem’ through injury surveillance or descriptive performance studies. The second stage aims to understand why and how injuries or performances occur, that is, identifying injury risk factors and performance determinants. The third stage seeks to develop potential preventive and performance enhancing measures for testing in ideal or controlled conditions (Stage four). Stage five aims to understand the implementation context and the final stage (Stage six) monitors the effectiveness of the preventive and performance measure in the real world.

The first stage is essential to these injury prevention and performance process models, as it provides direction and focus to the subsequent stages. A large proportion of RL injury and performance research falls within the first stage of the sequence. This has also shown to be the case in rugby union research.^[10]^ Literature reviews aim to search and synthesise this body of research with the goal of pushing forward the injury prevention and performance agenda, and to inform practice and coaching. While general epidemiology and performance reviews for RL have been conducted,^[4,11]^ a review specifically synthesising tackle injury epidemiology and tackle frequencies by playing levels, competition, and by role (ball carrier or tackler) has not been published. Therefore, to fulfil the first stage of TRIPP and AARMS, the purpose of this narrative synthesis was to systematically search and synthesise tackle injury epidemiology and tackle performance frequencies in RL.

## Methods

### Search strategy

A search was conducted for publications that reported tackle-specific factors in RL (rugby union and sevens were included in the search and analysed separately)^[10]^ according to systematic review guidelines.^[12,13]^ Three electronic databases (PubMed, Scopus and Web of Science) were searched using the keyword combinations ‘rugby’ AND ‘contact’, ‘rugby’ AND ‘tackle’, and ‘rugby’ AND ‘league’ AND ‘injur*’.

### Eligibility criteria

The search was limited to English-only articles published between January 1995 and October 2018. Articles that involved quantitative data on RL (including all ages and levels of play, and male and female players) were included. Only studies that included tackle-related match and/or training data (pertaining to tacklers, and/or ball carriers) specific to (1) injury epidemiology and (2) tackle performance frequencies were selected for inclusion. The included studies clearly defined and included the tackle or ball carrying as part of their analysis and did not group these aspects of play into general contact/collision data thus rendering them indistinguishable from other contact phases.

Rates for overall and time-loss tackle injuries (only studies reporting number of injuries per 1000 hours of exposure) and tackle-related injury frequencies (percentages) were tabulated (Tables 1–2). A time-loss injury is defined as an injury that resulted in a player being absent from normal match/training/recreational activities for seven days more (this definition was used in all studies reported in the current analysis but may differ for other studies) after the incident. Medical attention injuries are injuries that required treatment from a healthcare professional but resulted in no time away from normal activities. Overall injuries include both medical attention and time-loss injuries events. Injury case studies specific to tackle events were also reviewed (see Appendix in the supplementary material). Overall tackle numbers and rates (only studies reporting total number of tackles per match, and/or number of tackles per game or per minute) were tabulated (Table 3) and included in the current study. Narrative literature reviews, systematic reviews, meta-analyses, editorials, journal letters, book chapters, conference proceedings, discussions and qualitative research studies were excluded from the analysis. All other quantitative study types and case studies were accepted for review.

### Screening process

**Fig. 1:**
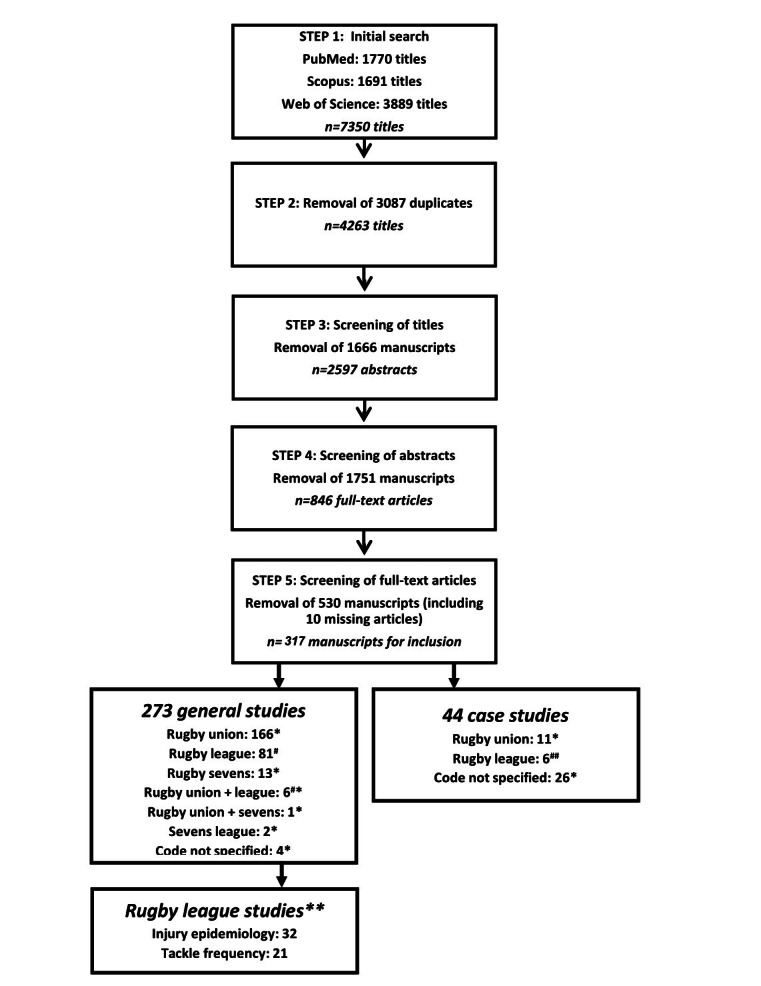
Summary of the literature screening process. ^#^, manuscripts included in RL analysis; ^##^, manuscripts included in RL appendix; *, manuscripts included in separate rugby union and sevens analysis/appendix; **, RL manuscripts included in the current study only.

A five-step approach was followed to identify the final group of articles that would eventually be included in the final review (Figure 1). Two authors (NB and SH) independently screened the titles using the eligibility criteria specified. The reliability of the authors was assessed by comparing the results of the title-screening process. Disparities in the results were discussed and resolved by the authors. NB continued the screening process of the abstracts and full-text articles. The articles were excluded at each step if they met the exclusion criteria or did not meet the inclusion criteria.

### Data interpretation

Only key findings (relating to tackle events) from each study were presented in this review. Confidence intervals (95% CIs) and standard deviations (±SDs) were provided in the tables (depending on availability). All values were rounded off to a maximum of two decimal places, where necessary.

## Results

The overall and time-loss injury frequencies and rates for professional RL are shown in Table 1 and all other levels in Table 2. Other RL tackle injury-related research also focused on specific types of injuries including concussion,^[14–20]^ spinal and neck injuries,^[21]^ and shoulder injuries.^[22]^ Tackle injury case-studies were also found and are summarised (see Appendix in the supplementary material).

The overall number of tackles per match and tackle rates (tackles per game or per minute) for all levels of play are shown in Table 3. Successful and unsuccessful tackles were grouped together.

## Discussion

The aim of this narrative synthesis was to systematically search for tackle injury and tackle frequency performance studies in RL and synthesise these with the goal of facilitating tackle injury prevention and performance research. This can be regarded as a reference document for coaches and practitioners. Based on the search criteria, a total of 53 studies were found: 32 focused on tackle injury epidemiology (with a further nine cases studies) and 21 focused on tackle frequency. In general, over 600 tackles may occur during a RL match. Each forward executes between 20–25 tackles, which is double that of backs. In contrast, forwards and backs carry the ball into the tackle, i.e. being tackled, at similar frequencies. A major challenge with the current body of literature on tackle frequencies in RL is the lack of or inconsistencies in defining the tackle. Studies have either used the definition of the tackle based on RL law, when the ball carrier is held by one or more of the opposing players, and either the ball or hand of the arm holding the ball makes contact with the ground, or the ball carrier cannot make any further progress, or it is used as a definition where ‘attempted’ tackles are included. To improve the consistency and quality of tackle research in RL, a set of clear descriptions and definitions need to be developed and agreed upon, similar to those used in rugby union.^[56]^

Tackle injury frequencies (both overall and time-loss injuries) ranged between 47%–94% at the professional level and between 38%–96% for the lower levels of play. Up to 73% of time-loss injuries, i.e. more severe injuries resulting in the player being absent from matches or training sessions taking place at least one week after the initial injury event, occurred during the tackle at the professional level.^[24]^ This frequency was lower at the amateur level, with up to 61% of time-loss injuries occurring during tackles.^[31]^ Also, the majority of time-loss and overall injuries occur to the tackled player (i.e. the ball carrier) in professional RL (63%) and across all other levels (61%). King et al.^[6]^ reported a high overall tackle-related injury incidence in a National Rugby League cohort (300 injuries per 1000 hours). This rate is substantially higher than the other figures reported in professional RL which were estimated at around 77.4 injuries per 1000 hours.^[26]^ However, overall injury rates are substantially higher in semi-professional cohorts, reaching up to 382.2 injuries per 1000 hours,^[32]^ and are even greater in amateur RL, with one study reporting incidences as high as 541 injuries per 1000 hours.^[39]^ It is worth noting that the majority of injury studies were conducted on single teams, which may explain why injury frequencies and incidences varied between studies. Although there were no explicit variations in the methods used to collect injury data across the studies, the differences in incidence rates may also be attributed to discrepancies in the levels of efficiency between the individual data capturers, although this cannot be confirmed.

This narrative synthesis was limited to the first stage of the TRIPP and AARMS models. As such, RL studies that are tackle-related but did not meet the criteria were excluded. For example, studies that focused on describing the types of tackles,^[2,48]^ tackle technique,^[5,57–62]^ or the physiological and physical loads associated with the tackle,^[43,47,48,51,52]^ were not included in the current review. Arguably, these studies relate to why and how injuries occur or what is required to succeed in the tackle, which fulfils the objectives of the second stage in the TRIPP and AARMS models, i.e. identifying injury risk factors and performance determinants. While an analysis of all tackle injury risk factors and performance determinants in RL was beyond the scope of the current synthesis, this paper forms the basis for such a review.

## Conclusion

This review synthesises tackle injury and tackle frequency performance studies in RL, with the goal of facilitating tackle injury prevention and performance research, and acting as a reference document for coaches and practitioners. Based on the search criteria, a total of 53 studies were found: 32 focused on tackle injury epidemiology and 21 focused on tackle frequency. In general, over 600 tackles may occur during a RL match. Each forward executes about 20–25 tackles, which is double that of backs. In contrast, forwards and backs carry the ball into the tackle, i.e. being tackled, at similar frequencies. Tackle injury frequencies (both overall and time-loss injuries) ranged between 47%–94% at the professional level, and between 38%–96% for the lower levels of play. A greater proportion of severe time-loss injuries occur during the tackle at the professional level in comparison to lower levels of play. Furthermore, most time-loss and overall injuries occur to players who are tackled, i.e. ball carriers, across all levels of play.

## Supplementary Information



## Figures and Tables

**Table 1:** Tackle-related injury frequencies and rates in professional Rugby League

Study	Cohort	Injury definition	Injury frequency	Injury rate
Gissane et al., 1998^[23]^	European Super League	Time-loss (>7 days)	70% (35% tackled, 35% tackling)	35.2/1000 hours (17.6/1000 hours tackled; 17.6/1000 hours tackling)
Gissane et al., 2003^[24]^	European Super League	Time-loss (>7 days)	73% (50% tackled, 23% tackling)	43.8/1000 hours (30.1/1000 hours tackled [95%CI 23.7–37.8]; 13.7/1000 hours tackling [95%CI 9.5–19.1])
Gissane et al., 2003^[25]^	European Super League	Overall and time-loss (>1 day)	74% (44% tackled, 30% tackling)	N/A
Gabbett et al., 2012^[26]^	National Rugby League	Overall and time-loss (>1 day)	N/A	77.4/1000 hours (30/1000 hours tackled [95%CI 22.6–39.7]; 47.4/1000 hours tackling [95%CI 38.0–59.1])
Georgeson et al., 2012^[27]^	National Rugby League	Overall and time-loss (>1 day)	49% (41% tackled, 8% tackling)	N/A
King et al., 2012^[6]^	National Rugby League	Overall and time-loss (>7 days)	94% (50% tackled, 44% tackling)	300/1000 hours (95%CI 265–340)
Sanctuary et al., 2012^[28]^	European Super League	Overall and time-loss (>1 day)	72%	N/A
Ullah et al., 2014^[29]^	National Rugby League	Overall and time-loss (>1 day)	47%	N/A

N/A, This data is not available for the study

**Table 2:** Tackle-related injury frequencies and rates in semi-professional, amateur and youth Rugby League

Study	Cohort	Injury definition	Injury frequency	Injury rate
Babic et al., 2001^[30]^	Croatian-Slovenian amateur rugby league	Overall and time-loss (>1 day)	55% (25% tackled, 30% tackling)	N/A
Gabbett, 2001^[31]^	Second Division amateur rugby league	Time-loss (>7 days)	61% (35% tackled, 26% tackling)	22.8/1000 hours (95%CI 12.0–33.7)
Gabbett, 2003^[32]^	Australian semi-professional rugby league	Overall and time-loss (>1 day)	46%	382.2/1000 hours (95%CI 355.5–408.9)
Gabbett, 2004^[33]^	Australian semi-professional rugby league	Overall and time-loss (>1 day)	38% (22% tackled, 16% tackling)	350.2/1000 hours (202.2/1000 hours tackled [95%CI 174.8–229.7]; 148.0/1000 hours tackling [95%CI 124.5–171.6])
Gabbett, 2005^[34]^	Australian semi-professional rugby league	Time-loss (>7 days)	53%	36.0/1000 hours (19.0/1000 hours tackled [95%CI 13.0–24.0]; 17.0/1000 hours tackling [95%CI 11.0–23.0])
Gabbett, 2005^[35]^	Sub-elite rugby league (^*^unlimited interchange)	Time-loss (>7 days)	N/A	37.4/1000 hours (19.8/1000 hours tackled [95%CI 12.3–27.2]; 17.6/1000 hours tackling [95%CI 10.5–24.6])
Gabbett, 2005^[35]^	Sub-elite rugby league (^*^limited interchange)	Time-loss (>7 days)	N/A	37.5/1000 hours (24.0/1000 hours tackled [95%CI 12.2–35.7]; 13.5/1000 hours tackling [95%CI 4.7–22.3])
Gabbett and Domrow, 2005^[36]^	Australian semi-professional rugby league	Time-loss (>7 days)	54% (30% tackled, 24% tackling)	29.7/1000 hours (16.5/1000 hours tackled [95%CI 12.1–20.9]; 13.2/1000 hours tackling [95%CI 9.3–17.1])
King et al., 2006^[37]^	New Zealand semi-professional rugby league	Overall and time-loss (>1 day)	66% (43% tackled, 23% tackling)	242.7/1000 hours (158.3/1000 hours tackled [95%CI 78.2–238.4]; 84.4/1000 hours tackling [95%CI 25.9–142.9])
King et al., 2006^[37]^	New Zealand amateur rugby league amateur	Overall and time-loss (>1 day)	68% (44% tackled, 24% tackling)	483.1/1000 hours (310.6/1000 hours tackled [95%CI 167.1–454.0]; 172.5/1000 hours tackling [95%CI 65.6–279.5])
Gabbett, 2008^[38]^	Under-19 rugby league	Time-loss (>7 days)	N/A	29.3/1000 hours (19.2/1000 hours tackled [95%CI 11.0–27.5]; 10.1/1000 hours tackling [95%CI 4.1–16.0])
King et al., 2009^[39]^	Division 1 Premier Grade amateur rugby league	Overall and time-loss (>1 day)	86% (overall) (48% tackled, 37% tackling); 87% (time-loss) (51% tackled, 36% tackling)	228.1/1000 hours (overall) (128.5/1000 hours tackled [95%CI 103.2–160.0]; 99.6/1000 hours tackling [95%CI 77.7–127.8]); 54.6/1000 hours (time-loss) (32.1/1000 hours tackled [95%CI 20.7–49.8]; 22.5/1000 hours tackling [95%CI 13.3–38.0])
King et al., 2009^[39]^	Division 2 Premier Grade amateur rugby league	Overall and time-loss (>1 day)	77% (overall) (58% tackled, 19% tackling); 71% (time-loss) (44% tackled, 27% tackling)	541.0/1000 hours (overall) (404.9/1000 hours tackled [95%CI 338.3–484.5]; 136.1/1000 hours tackling [95%CI 99.8–185.5]); 153.1/1000 hours (time-loss) (95.3/1000 hours tackled [95%CI 65.8–138.0]; 57.8/1000 hours tackling [95%CI 36.0–93.0])
King et al., 2010^[7]^	Premier division amateur rugby league	Overall and time-loss (>1 day)	96% (68% tackled, 28% tackling)	501.3/1000 hours (356.7/1000 hours tackled [95%CI 258.4–492.3]; 144.6/1000 hours tackling [95%CI 87.2–239.8])
Orr and Cheng, 2016^[40]^	Elite Australian junior rugby league	Overall and time-loss (>1 day)	61% (overall) (36% tackled, 25% tackling); 66% (time-loss) (42% tackled, 24% tackling)	N/A

N/A, This data is not available for the study

**Table 3:** Tackle numbers and rates in Rugby League (all levels of play)

Study	Cohort	Rate definition	Tackle rate
Sirotic et al., 2009^[41]^	National Rugby League	Tackling/minute	0.25 (SD±0.16) (1^st^ half: 0.26 [SD±0.18]; 2^nd^ half: 0.23 [SD±0.16])
Sirotic et al., 2009^[41]^	Semi-professional New South Wales Premier League	Tackling/minute	0.28 (SD±0.16) (1^st^ half: 0.30 [SD±0.17]; 2^nd^ half: 0.27 [SD±0.16])
King et al., 2010^[2]^	Elite rugby league (international, NRL, national youth competition)	Total match tackles (tackling and tackled) Tackling/game Tackled/game	701 (SD±64)a 32 (SD±15)b 21 (SD±4)c
Gabbett et al., 2011^[42]^	National Rugby League	Total match tackling attempts Tackling/game	265.1 (SD±214.1)^a^ 17.1 (SD±9.1)b
McLellan et al., 2011^[43]^	National Rugby League	Tackling/game Tackled/game	14.9 (SD±10.5)^a^ (forwards: 20.1 [SD±11.3]; backs: 10.7 [SD±8.0]) 10.2 (SD±3.8)^b^ (forwards: 10.9 [SD±4.2]; backs: 9.7 [SD±3.5])
Sirotic et al., 2011^[44]^	National Rugby League	Tackling/minute	Backs: 0.12 (SD±0.09); Forwards: 0.41 (SD±0.07); Fullbacks: 0.05 (SD±0.02); Hookers: 0.34 (SD±0.11) Service: 0.31 (SD±0.12)
Sykes et al., 2011^[45]^	English Super League and National Rugby League	Tackling/minute Tackled/minute	1^st^ quarter: 0.28 (SD±0.21)^a^; 0.14 (SD±0.10)^b^ 2^nd^ quarter: 0.28 (SD±0.27)^a^; 0.13 (SD±0.12)^b^ 3^rd^ quarter: 0.26 (SD±0.22)^a^; 0.13 (SD±0.10)^b^ 4^th^ quarter: 0.32 (SD±0.33)^a^; 0.14 (SD±0.09)^b^
Duffield et al., 2012^[46]^	Amateur rugby league	Tackles (tackling and tackled)/game	22 (SD±9)
King et al., 2012^[6]^	Professional rugby league	Total match tackles (tackling and tackled)	657
McLellan and Lovell, 2012^[47]^	National Rugby League	Tackling/game Tackled/game	19.9 (SD±10.5)^a^ (forwards: 26.1 [SD±15.3]; backs: 10.7 [SD±8.9]) 12.2 (SD±3.6)^b^ (forwards: 13.8 [SD±5.2]; backs: 11.7 [SD±4.6])
Twist et al., 2012^[48]^	European Super League	Tackling/game Tackling/minute Tackled/game Tackled/minute	Forwards: 25.5 (SD±13.7)^a^; 0.5 (SD±0.2)^b^; 12.7 (SD±6.1)^c^; 0.3 (SD±0.1)^d^ Backs: 13.6 (SD±7.9)^a^; 0.2 (SD±0.1)^b^; 11.6 (SD±3.4)^c^; 0.1 (SD±0.04)^d^
Johnston et al., 2013^[49]^	Amateur rugby league	Tackling/minute	0.3 (SD±0.1)
McGuckin et al., 2014^[50]^	National Rugby League	Tackling/game	Home games: 14.3 (SD±6.7); away games: 18.4 (SD±9.2)
Cummins and Orr, 2015^[51]^	National Rugby League	Tackling/game Tackling/minute Tackled/game Tackled/minute	Hit-up forwards: 21.5 (SD±6.0)^a^; 0.53 (SD±0.08)^b^; 9.0 (SD±3.8)^c^; 0.20 (SD±0.04)^d^ Wide-running forwards: 20.5 (SD±5.0)^a^; 0.39 (SD±0.10)^b^; 8.0 (SD±3.6)^c^; 0.20 (SD±0.10)^d^ Adjustables: 17.0 (SD±12.5)^a^; 0.41 (SD±0.20)^b^; 5.0 (SD±4.5)^c^; 0.1 (SD±0.00)^d^ Outside backs: 7.0 (SD±6.0)^a^; 0.08 (SD±0.07)^b^; 11.2 (SD±2.1)^c^; 0.1 (SD±0.02)^d^
Fletcher et al., 2015^[52]^	European Super League	Tackling/game Tackled/game	Forwards: 24 (SD±13)^a^; 8.5 (SD±5)^b^ ; Backs: 8 (SD±10)^a^; 9 (SD±4)^b^ Adjustables: 14 (SD±12)^a^; 4 (SD±4)^b^
Speranza et al., 2015^[53]^	Semi-professional rugby league	Tackling/game	Forwards: 24.3 (SD±6.5); Backs: 13.2 (SD± 8.5)
Gardner et al., 2017^[17]^	National Rugby League	Total match tackles (tackling and tackled)	632.1
Dempsey et al., 2018^[54]^	Senior international male rugby league	Tackling/game Tackling/minute Tackled/game Tackled/minute	Forwards: 25.5 (SD±8.4)^a^; 0.47 (SD±0.23)^b^; 10.5 (SD±3.6)^c^; 0.20 (SD±0.10)^d^ Backs: 13.4 (SD±9.5)^a^; 0.16 (SD±0.11)^b^; 11.9 (SD±5.2)^c^; 0.15 (SD±0.08)^d^
Dempsey et al., 2018^[54]^	Junior international male rugby league	Tackling/game Tackling/minute Tackled/game Tackled/minute	Forwards: 19.2 (SD±10.0)^a^; 0.34 (SD±0.13)^b^; 6.5 (SD±3.5)^c^; 0.12 (SD±0.06)^d^ Backs: 10.0 (SD±6.7)^a^; 0.13 (SD±0.08)^b^; 5.3 (SD±3.5)^c^; 0.06 (SD±0.04)^d^
Woods et al., 2018^[55]^	National Rugby League	Total match tackle attempts	325.0 (SD±39.7)
Woods et al., 2018^[55]^	Under-20 Holden Cup	Total match tackle attempts	283.4 (SD±35.6)
