# Supplementary material for: Tackle injury epidemiology and performance in rugby league – narrative synthesis
Source: S Afr J Sports Med. 2021 Mar 2;33(1):v33i1a9313. doi: 10.17159/2078-516X/2021/v33i1a9313 (PMC9924599; doi:10.17159/2078-516X/2021/v33i1a9313)
Supplement: Supplementary file 1 [file 2078-516X-33-v33i1a9313-s001.pdf]

Tackle-related injuries in RL case studies

Study	Player details	Injury mechanism	Diagnosis	Treatment and long-term outcome
Geffen et al., 1997[1]	22-year-old male professional rugby league player	Tackled opponent with left shoulder in midriff with back slightly flexed, knocked to ground (tackler)	Complex thoracic vertebrae injury (fracture of T6 interarticular processes, compression fractures to bodies of T6 and T7, inferior facet fracture of T6)	Conservative management, returned to contact sports 6 months after injury
Pollard et al., 2005[2]	30-year-old male professional rugby league player (front-row)	Hyperflexion and neck compression as a result of being tackled (whiplash associated disorder or WAD), same event repeated on several occasions over 4 months	Decreased disc height at C5-C6/C6-C7 levels, calcification within anterior longitudinal ligament at C6-C7 level, right postero-lateral disc protrusion at C6-C7 level	Manipulative therapy, proprioceptive neuromuscular facilitation (PNF) stretching, soft tissue massage, trigger point therapy, retired from sport
Shillington et al., 2008[3]	23-year-old male rugby league player	Tackled by 3 opponents, landed on knee, valgus and hyperflexion force under weight of 2 defenders (tackled)	Rupture of patellar tendon, anterior cruciate and medial collateral ligaments, with medial meniscal tear	Two surgeries with rehabilitation period in between, straight line running 4 months after second surgery and rehabilitation
Kolar et al., 2011[4]	37-year-old male rugby league player	Right foot stuck in mud, tackled from behind, pelvis and flexed hip forced to twist (tackled)	Luxatio erecta (inferior dislocation) of hip	Relocation of dislocated hip, returned to contact sports 6 weeks after injury
Sullivan et al., 2017[5]	20-year-old male professional rugby league player	Experienced external rotation and direct eversion of fixed right ankle during a tackle (tackled)	Oblique fibula fracture, complete rupture of anterior tibiofibular ligament (ATFL), calcaneofibular ligament (CFL), and deep and superficial fibres of deltoid ligament, as well as a syndesmotic injury with complete rupture of	Surgery, conservative management, returned to contact sports after rehabilitation 24 weeks after injury

			anterior inferior tibiofibular ligament (AITFL) and posterior inferior tibiofibular ligament (PITFL)	
Koong et al., 2018[6]	22-year-old male professional rugby league player	Sustained lateral blow to fixed, semi-flexed left knee while running (tackled)	Complete tear of popliteus tendon at musculo-tendinous junction with retraction to popliteus hiatus as well as oedema around muscle belly	Conservative management, returned to contact sports after rehabilitation 4 weeks after injury

References

1. Geffen S, Gibbs N, Geffen L. Thoracic spinal fracture in a rugby league footballer. *Clin J Sport Med.* 1997;7(2):144–146.

2. Pollard H, Hansen L, Hoskins W. Cervical stenosis in a professional rugby league football player: a case report. *Chiropr Osteopat [Internet].* 2005;13(15):1–6. Available from: http://www.pubmedcentral.nih.gov/articlerender.fcgi?artid=1185557&tool=pmcentrez&rendertype=abstract

3. Shillington M, Logan M, Watts M, Myers P. A complex knee injury in a rugby league player. Combined rupture of the patellar tendon, anterior cruciate and medial collateral ligaments, with a medial meniscal tear. *Inj Extra [Internet].* 2008;39(10):327–328. Available from: http://dx.doi.org/10.1016/j.injury.2008.05.004

4. Kolar M, Joseph S, McLaren A. Luxatio erecta of the hip. *J Bone Jt Surg.* 2011;93:273.

5. Sullivan M, Fraser EJ, Linklater J, Harris C, Morgan K. Arthroscopic Surgical Technique for an Acute Talar Dome Osteochondral Lesion in a Professional Rugby League Player. *Foot Ankle Spec.* 2017;10(3):263–269.

6. Koong DP-H, An VVG, Lorentzos P, Moussa P, Sivakumar BS. Non-Operative Rehabilitation of Isolated Popliteus Tendon Rupture in a Rugby Player. *Knee Surg Relat Res.* 2018;30(3):269–272.
